# Vision Transformer attention alignment with human visual perception in aesthetic object evaluation

**DOI:** 10.1371/journal.pone.0344006

**Published:** 2026-04-03

**Authors:** Miguel Carrasco, César González-Martín, José Aranda, Luis Oliveros

**Affiliations:** 1 School of Computer Science and Telecommunications, University fo Diego Portales, Santiago, Chile; 2 Faculty of Education and Psychology, University of Cordoba, Cordoba, Spain; 3 School of Computer Science and Telecommunications, University of Adolfo Ibáñez, Santiago, Chile; Xidian University, CHINA

## Abstract

Visual attention mechanisms play a crucial role in human perception and aesthetic evaluation. Recent advances in Vision Transformers (ViTs) have demonstrated remarkable capabilities in computer vision tasks, yet their alignment with human visual attention patterns remains underexplored, particularly in aesthetic contexts. This study investigates the correlation between human visual attention and ViT attention mechanisms when evaluating handcrafted objects. We conducted an eye-tracking experiment with 30 participants (9 female, 21 male, mean age 24.6 years) who viewed 20 artisanal objects comprising basketry bags and ginger jars. Using a Pupil Labs eye-tracker, we recorded gaze patterns and generated heatmaps representing human visual attention. Simultaneously, we analyzed the same objects using a pre-trained ViT model with DINO (Self-DIstillation with NO Labels), extracting attention maps from each of the 12 attention heads. We compared human and ViT attention distributions using four complementary metrics—Kullback-Leibler divergence, Structural Similarity Index (SSIM), Pearson’s Correlation Coefficient (CC), and Similarity (SIM)—across varying Gaussian parameters (σ=0.1−4.0), yielding 1,152,000 distance evaluations. Additionally, we performed Areas of Interest (AOI) analysis to quantify ViT attention concentration within object regions. Statistical analysis revealed optimal correlation at σ=2.4±0.03, with attention head #12 showing the strongest alignment with human visual patterns across all metrics. Significant differences were found between attention heads, with heads #7 and #9 demonstrating the greatest divergence from human attention (p≤0.05), Tukey HSD test). AOI analysis confirmed that all ViT heads concentrated attention significantly more within object regions than background areas (p≤0.0001), with heads #12, #1, and #3 achieving lift values of +30 to +40 percentage points. Results indicate that while ViTs exhibit more global attention patterns compared to human focal attention, certain attention heads can approximate human visual behavior, particularly for specific object features like buckles in basketry items. These findings suggest potential applications of ViT attention mechanisms in product design and aesthetic evaluation, while highlighting fundamental differences in attention strategies between human perception and current AI models.

## Introduction

Human visual attention is a crucial process that allows individuals to focus on specific visual stimuli, filtering information from the environment, necessary due to the biological limitations of processing all the visual inputs we receive [1], which is essential for human perception [2] and affects their behavior [3,4]. Before attention, preattention occurs, a selective attention where some inputs are weighted over others, and the weights must be chosen for specific objectives [5]. For this, an analysis of visual characteristics [6] or low-level features (e.g., color, shape, orientation) [7], and their location in space [8], also called mid-level features [9,10], takes place. Without instruction to the observer, the contrast between the visual characteristics of an object and the other components of the scene appears to be determinant in guiding attention [11]. In short, singularities in images are the characteristics that determine visual attraction [12]. Visual attention is composed of two types of mechanisms: overt, moving the eyes toward a specific object, and covert, which is when attention is focused on a peripheral zone, voluntarily or involuntarily, without directing the gaze there, with the latter preceding the former [13,14]. In turn, visual attention is categorized into two functions: Bottom-up, which is initial attention produced by salient stimuli in the environment, and top-down attention, which is captured by the relevance, objectives, intentions, context, and prior knowledge of the observer [2,15–17]. However, this dichotomy is debated [18] as they are two neurocorrelated processes [15].

Visual attention processing requires sustained effort to maintain focus on a stimulus, occurring through both intentional and automatic mechanisms [19]. Contrary to common assumptions, visual attention operates as a slow, rhythmic process [20] that varies depending on the object’s location within the representational space [21]. This variability is evident in findings showing that the initial fixation does not determine the subsequent course of action [22]. The importance of observation time on an object has been studied as a strong predictor of purchase [23–25], and it is deduced that the more you like an object, the longer you look at it, which increases the possibility of purchase [26]. However, while some theoretical currents maintain that the purchase decision occurs after fixations, others point out that this action takes place during fixations [27]. In this sense, aesthetics plays an important role in visual exploration [28] through visual characteristics, such as orientation, luminance, size, color, or shapes, positively influencing the speed of visual search [29], capturing and preserving visual attention more effectively [30] and therefore, fixations, which improves perception and is related to emotions [31,32], regardless of conditions [33] and the nature of the object [13,34]. The correlation between visual attention and aesthetic preference has been studied through faces [35], objects [36], architecture [37], or works of art [38], demonstrating that it affects self-relevance [39]. However, to the extent of our knowledge, visual attention in artisanal production has not been explored in depth, where the aesthetic dimension is also a determining factor for its consumption. One could cite the work of S. Zhang [40], who shows the aesthetic influence of plates on food

In visual attention analysis, eye tracking technologies have emerged as the predominant technique in recent years, driving extensive research and development across diverse fields [41,42]. These technologies have proven particularly valuable for studying artistic objects [43,44] across various typologies and styles [13,35,45–47], establishing themselves as an ideal tool for visual attention research [42]. However, the recent emergence of the Deep Learning model called Vision Transformers (ViT) has revolutionized the field of computer vision and automatic image processing, equaling or surpassing other computational models such as Convolutional Neural Networks (CNNs) by using image patches and attributing positional embeddings to them, passing through the encoder independently, which allows it not to lose information about their order [48]. Within the encoder, these patches pass through an attention module that contains multi-head attention layers that achieve the so-called self-attention characteristic of ViT. Its peculiar structure gives it a variety of unique characteristics, highlighting the ability to incorporate global and local information in the lower layers of the network [49]. Additionally, they manage to create shortcuts between their neurons that facilitate connections and performance, allowing them to have an understanding of the complete context of the image and, from the beginning, can classify even when the image pieces are not delivered in the correct order, unlike CNN models, which depend on initial layers focused only on local information. This self-attention mechanism was originally proposed in the Transformer model by Vaswani et al. [50], allowing simultaneous relation of all input regions at different levels of spatial hierarchy. However, it cannot be well explained how ViT determines the attention of each part of the images it classifies. Understanding this depends on a large number of neurons in the model, and a black box effect occurs, where it is impossible to see the steps taken to reach the result. Recent studies have observed that, although Vision Transformers manage to capture perceptual groupings similar to humans, they tend to assign relevance differently, sometimes highlighting distractors or secondary elements [51].

Studies such as Raghu et al. (2021) [49] have delved into understanding the functioning of ViTs compared to CNN models. Given the difficulty, the study was based on Central Kernel Alignment (CKA), which provides a scalar value that can be used to determine quantitative similarity between different layers more easily. When applying CKA to ViT and ResNet, it was determined that their first 60 layers were similar, but later in the upper layers, they differed considerably. Additionally, ViT layers change uniformly while ResNet layers have an abrupt change between lower and upper layers. The functioning of multi-head attention layers was also analyzed by restricting the distances they covered. In this way, it was discovered that they provide global information even in the lower layers, which differed completely from CNNs, where the first layers contain only local information. Even when implementing tokens to ResNet that represent convolutional channels of a particular spatial zone to compare their functioning with ViT attention, it was observed that these focus better on the image and its contour compared to CNNs that use more of the image’s texture for classification. Despite the above, they were able to discover several characteristics of how self-attention functions. They noted that their methodology, based on the use of CKA could be deepened with finer methods. In other studies such as Tuli et al. (2021) [51], they have delved into the problem using other metrics. In this case, precision and error when classifying the same set of images, they found that ViTs are more similar to humans than CNNs. Even so, a new perspective on the internal functioning of ViT attention could not be provided. On the other hand, there is the possibility of deepening knowledge of multi-head attention layers through analysis of their attention and working inversely, from the result in images toward the internal structure of these. Due to this, it is appreciated that the flexibility of self-attention in ViT is closer to human vision. In this aspect, the study agrees that ViT better explains human visual attention during reading than the computational E-Z Reader model.

Conversely, studies that demonstrate gaps between traditional/CNN-based saliency models, deep neural networks, and human performance in visual processing show that ViTs tend more toward perceptual grouping than attention, which approximates the behavior of lateral interactions in the human visual cortex [52]. On the other hand, Mehrani and Tsotsos (2023) [53] demonstrate that ViTs assign relevance to elements differently from human attention, highlighting distractors or elements located in the background in the results. Along this line, they point out that human visual attention involves both feed-forward and feedback mechanisms, while in ViTs, feed-forward mechanisms predominate, suggesting fundamental differences in how attention is implemented. It has been proposed that a key difference between human attention and that of ViTs lies in the combination of feedforward and feedback mechanisms in humans, while in ViTs a primarily feedforward architecture predominates, limiting their approximation to natural visual processing, performing more global attention [54].

Given the conflicting findings regarding the similarity between ViT and human attention mechanisms, this research investigates the correlation between these two attention types when participants view identical samples of artisanal objects. We selected two distinct categories: bags created through basketry techniques and ginger jars. This choice builds upon established research using artistic objects in attention studies, leveraging their rich visual characteristics and aesthetic properties for analysis.

The selection of these two object types is strategically designed to provide morphological contrast. Basketry bags exhibit predominantly rectilinear forms with polyhedral structures, while ginger jars feature vertical orientation with curvilinear shapes. This deliberate contrast in form and structure enables us to examine how different visual characteristics influence attention patterns and detect variability in attentional responses across object categories. The study of similarity between the two attentions (human vs ViT) will allow for deeper exploration of the use of this technology in the creation and design process of commercial products, for detecting visual attraction zones, thus allowing knowledge of the level of visual attraction in advance. Therefore, this research establishes the following hypotheses:

- Hyp.1. The Vision Transformer attention module and human visual attention do not present statistically significant differences
- Hyp.2. ViT is an applicable technology in artisanal product design for detecting aesthetic interest zones.

To respond to the proposed hypotheses, the following objectives are established:

- O1. Statistically determine the correlation between ViT and human attention in a dataset of images of artisanal products
- O2. Analyze visual interest zones in artisanal objects with both attention mechanisms (ViT and human)

## Materials and methods

**Fig 1:**
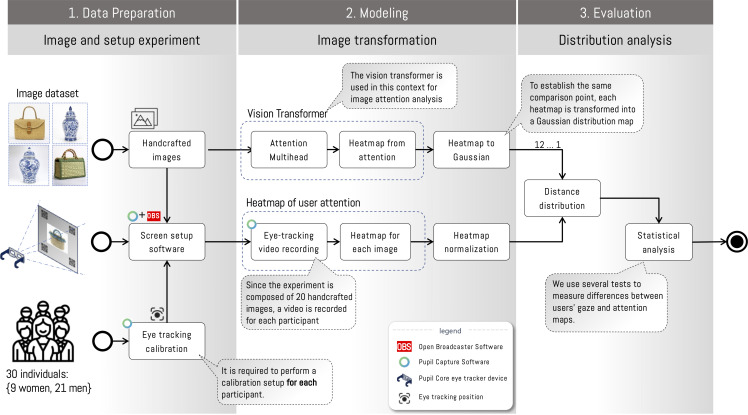
General diagram of the experimental attention analysis process. The process is composed of three stages: 1) data preparation and experimental setup, 2) image transformation, and 3) distribution analysis. Data preparation and setup consist of the image evaluation process by each experiment participant. This stage requires the use of an eye-tracker to determine participants’ gaze positions and define the experimental conditions. Image transformation consists of generating an attention map using the ViT attention module and of having users experimentally evaluate it on a set of objects. The last stage compares both information sources and thus determines whether there is any correlation. *Note: The craft figures shown are similar but not identical to the original images and are included for illustrative purposes only*.

The methodology is composed of three stages defined as data preparation, modeling, and evaluation. Each of them is composed of sub-components that allow the integration of the experiment through the flow of information between software and experimental components (see Fig 1). Below, we explain each of the stages in detail.

### Data preparation

**Fig 2:**
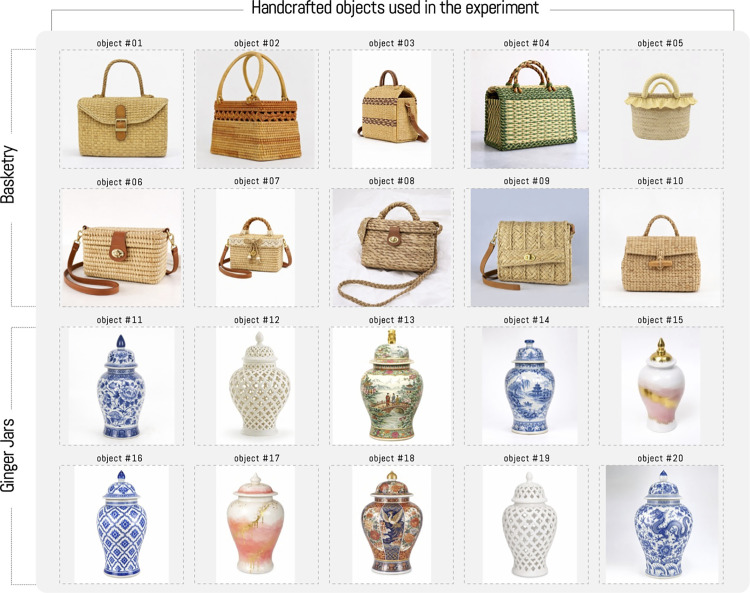
Objects used in the experiment consisted of ten basketry objects and ten ginger jars. The objects were randomly selected, with the fewest possible objects in the background. *Note: The craft figures shown are similar but not identical to the original images and are included for illustrative purposes only*.

The experiment consists of having a set of people view a group of images in a controlled environment using an eye-tracker. The analyzed objects correspond to craft pieces, specifically basketry and ginger jars. The selection comprised 10 basketry objects and 10 jars, which could be seen clearly and without relevant external visual distractors (see Fig 2). The selected objects vary slightly in size and decoration, maintaining unity in their materials, colors, and shapes to reduce distractions and draw attention to details. In the case of the jars, these have curvilinear forms and common structures, but with variations mainly highlighted in decorations (colors, figures and shapes). In both cases, scene distractors have been reduced and all are free of logos and text.

**Fig 3:**
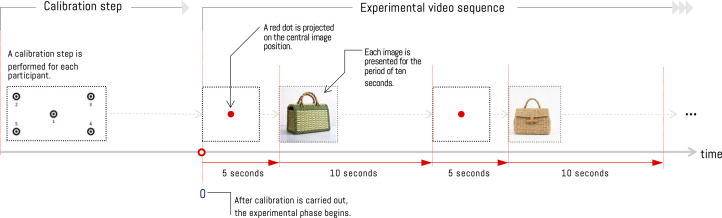
Experimental procedure for object visualization. Before starting the experimental phase, a calibration procedure is performed with the recording of a sequence of five points on the screen. Once this process is completed, the experimental phase begins with the projection of an image with a white background and a red dot, displayed for 5 seconds. Then one of the 20 objects is displayed for 10 seconds. This procedure repeats until all objects have been displayed.*Note: The craft figures shown are similar but not identical to the original images and are included for illustrative purposes only*.

To record visual information from participants, we have used a Pupil Core eye tracker from Pupil Labs via the Pupil Capture software. This software allows recording from multiple device sensors, such as a microphone, a front camera, and pupil refraction cameras. Additionally, the software performs camera synchronization for eye-tracking calculations during a calibration process (Fig 3, calibration step). This process allows precise determination of the user’s gaze on the experiment screen. To do this, it relates the gaze position on the screen with the eye position relative to the eye-tracker’s internal camera.

The image sequences displayed were generated using the following procedure. First, a red dot is presented at the center of the screen for five seconds. Then each of the 20 objects is presented for ten seconds. Then it returns to the first step to transition, object by object, from the basketry set to the jars (Fig 3). The objective of looking at a red dot during the transition between objects is to center the gaze at the same position when a new object is displayed. In this way, we reduce gaze-position error by being in a different position during the transition. On the other hand, displaying the image for 10 seconds was chosen because it falls within a time range that allows human attention to focus on the most relevant information, reduces fixation driven by mental load, and provides a sufficient number of effective fixation samples cite locher2007visual.

**Fig 4:**
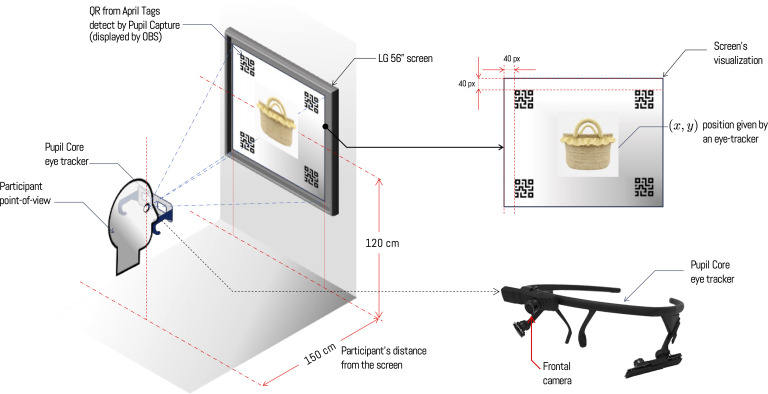
Participant setup in front of the screen during the experimental phase. All participants remain seated while the experiment is conducted. At the beginning of each experiment, a calibration process is performed with the eye-tracker and the experiment is explained to the participant. The chosen distance between the user and screen remains relatively fixed at 150 cm, as it reduces visual fatigue. *Note: The craft figures shown are similar but not identical to the original images and are included for illustrative purposes only*.

The experiment uses a reference system that allows real-time location of gaze position on the screen. For this, it is necessary to employ four QR markers of the April Tags type, which are recognized in real time by the Pupil Capture software and enable precise determination of gaze position relative to a reference system. This allows participants to move their bodies and heads freely, and the software detects gaze position relative to the screen. This procedure has been carried out in combination with OBS (Open Broadcaster Software), which displays a QR code in each corner of the screen, along with object display through an application developed in Python. On the other hand, the distance between the screen and the user has been maintained at 150 cm, as it allows covering a large part of the participant’s visual field and minimizing visual fatigue (see setup in Fig 4). This procedure has been implemented on an Asus TUF 15 computer with 16GB of RAM, Intel Core i5 CPU and an NVIDIA 1650 graphics card, and images have been projected on an LG screen with 56-inch resolution and 60 Hz refresh rate.

To obtain statistically valid results, the project has collected data from 30 participants who voluntarily participated in the experiment and signed informed consent. The selected participants meet the following criteria: 1) being persons over eighteen years of age, 2) not reporting any pathology or ocular deficiency that would prevent them from viewing images at distances less than 150 cm away, and 3) not reporting any type of pupil refraction that would make it unfeasible to use an eye-tracker device with this type of technology. An exception was made if the condition can be corrected through contact lenses, as these do not alter the measurements of the eye tracker used. Additional details were recorded for all participants, including age, gender, and area of knowledge or profession, to analyze whether these factors are related to each individual’s visual attention. The recruitment period for the study occurred from 22 to 26 January 2024. This research was conducted with the approval of the ethics committee of Universidad Adolfo Ibáñez (certificate 57/2023).

### Modeling

**Fig 5:**
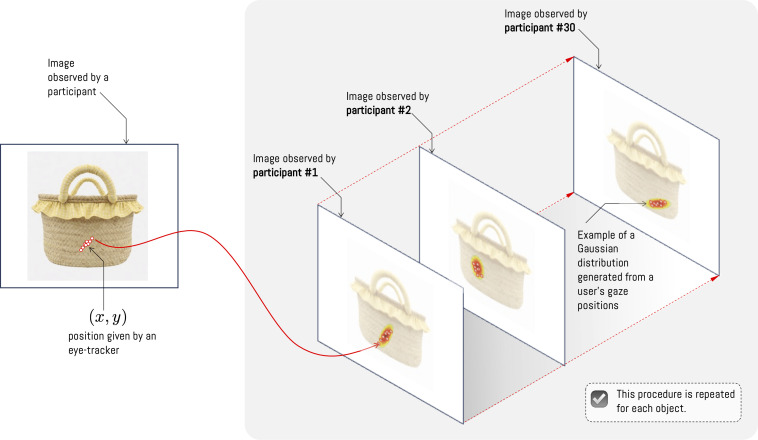
Heatmap generation according to positions recorded by each observer. The heatmap of each object is constructed as the average of individual visualizations transformed to a two-dimensional Gaussian distribution. *Note: The craft figures shown are similar but not identical to the original images and are included for illustrative purposes only*.

The data generated by the procedure described in Fig 3 are processed using Pupil Player Software, allowing obtaining the gaze position of each participant, which are expressed as coordinates (*x*,*y*) in relation to the viewing area (Fig 5). Additionally, it is possible to obtain the timestamp, the corresponding frame in the recording, the pupil position coordinates, and the measurement confidence level. Thanks to the time and gaze-position information, it is possible to estimate a Gaussian distribution for each coordinate, taking into account the relative time spent at each location, which allows generating a heat map. The longer the gaze remains on a certain position in the image, the density in that region increases (see example in Fig 5). This process is performed by each participant on the set of images, yielding a set of Gaussian distributions for each image; each distribution is independent of the results of other observations. In this way, the distributions are considered independent, and their averages can be taken as the results of observations for each image. Finally, to compare this result with the Vision Transformer attention module, the distributions for each experiment image are normalized.

For Vision Transformer (ViT) training, the pre-training available in Facebook Research’s DINO (Self-Distillation with NO Labels) repository has been used. This research uses a training scheme that consists of training a ViT using another in a teacher-student relationship. Specifically, a process known as self-distillation is applied in which two ViTs pre-trained with the same dataset without categories (ImageNet) but with different parameters are used. The same images are passed through each one, with the difference that for the student, they are segments of at most 50% of the image, while for the teacher, they are equal to or greater than 50%. Then, the teacher ViT weights are updated using the student’s weights via the Exponential Moving Average (EMA) technique, and the teacher is then centered using a batch mean to avoid dominance by any learned feature. Finally, the softmax function is applied to compute the cross-entropy between the two networks, ensuring they maintain the same distribution. This form of training achieves better performance and classification power than supervised training, in addition to superior segmentation of relevant zones, which provides clearer visual contrast for human attention [55].

The architecture used generates 12 independent distributions, each stored in one head of the attention module. In this way, it is possible to either consider the average of the 12 heads (similar to the analysis applied to participants) or treat each distribution as an independent result. To visualize the attentions generated by the ViT, specific functions were added to extract the weights from each attention head. As with the attention transformation process applied to participants, we have converted each weight from the 12 heads into Gaussian distributions to analyze the differences between the two.

To visualize the attentions generated by the ViT, we extract the last-layer self-attention and retain the [CLS]→patch slice for each of the 12 heads. The resulting vector with N=(H/p)×(W/p) elements is reshaped into the patch grid and upsampled to pixel resolution with nearest-neighbour using a scale factor equal to the patch size (*p* = 16). Inputs are cropped to the nearest multiple of *p* to preserve patch alignment. ViT attention maps are min–max normalized to sum to one before comparison.

### Evaluation

To analyze the differences between the heat maps generated by ViT attention and the participants’ average visualization, we evaluate multiple distances between distributions. In particular, we use the Kullback–Leibler divergence (KL), the Structural Similarity Index (SSIM), Pearson’s Correlation Coefficient (CC), and Similarity (SIM). Below, we briefly describe each metric: (1) The Kullback–Leibler divergence, also called relative entropy, is a distance between distributions that is defined relative to one of them; that is, one distribution is used as a reference point [56]. A simple percentage-difference calculation can be more biased toward distributions with larger values, and, therefore, Kullback–Leibler applies a logarithmic function to reduce this bias. It is then multiplied by the distribution used as a reference point to apply the difference to each variable of the distribution. (2) The Structural Similarity Index (SSIM) is a metric used to compare images by considering how the human eye perceives them. Unlike measures based only on pixel-by-pixel error, SSIM evaluates similarities in luminance, contrast, and structure. This enables the detection of visual degradations that other metrics do not capture well. For this reason, SSIM is widely used because it provides a measure that is more consistent with perceived visual quality [57]. (3) Pearson’s Correlation Coefficient (CC) is used to measure the degree of linear similarity between two images, evaluating how their pixel intensities vary jointly. Its value ranges from −1 to 1, where values close to 1 indicate high similarity, 0 indicates no correlation, and −1 indicates an inverse relationship. CC is useful for comparing global intensity patterns, although it does not capture local differences or subtle structural changes well [58]. (4) Similarity (SIM) is a metric used to compare two normalized maps or images by evaluating how much their distributions overlap. It is computed as the minimum value across both maps at each pixel, making it intuitive and easy to interpret. A high value indicates that the two representations share similar patterns, whereas a low value reflects clear discrepancies. SIM is especially common in saliency-map evaluation because it captures global agreement without excessively penalizing small local differences [58]. Although other metrics have been reported in the literature, we consider that the metrics above cover a large part of the analysis related to saliency comparisons.

The objective is to determine whether there is any head whose gaze distance is similar to that of one of the attention module’s heads. For this, we use a statistical test that measures the difference between medians, which requires that the samples be independent, continuous, and of the same size. To determine which heads differ, the Tukey Honestly Significant Difference (HSD) test was applied as a post hoc test to each possible head pair at the same significance level.

To identify atypical heads, we have determined the p-values, identified the heads that achieved the lowest values in each combination, and determined whether they did not exceed the null hypothesis using the HSD test. In this way, it is possible to determine if the null hypothesis is refuted and, therefore, there is at least one different distribution.

### Statistical analysis of Areas of Interest (AOI)

To quantify whether the ViT attention heads concentrate activation in regions that are relevant to human participants, we compare, for each ViT head, the hit rate within the Areas of Interest (AOI) against that of regions outside the AOI. An AOI is a specific region with semantic meaning in the image, manually defined over the participant’s viewing area; its complement is referred to as *non-AOI*. We also consider *hits*: a hit is a fixation such that, for head *h*, the value of the ViT attention map at the corresponding pixel exceeds a threshold τ set per image and per head. The dataset contains images from two object groups (*basketry* and *jar*). For each image, we have fixation records from 30 participants and their corresponding AOI masks. The attention maps come from a pretrained ViT: for each head, we extract the attention from the [CLS] token to the *patch* tokens and interpolate it to the original image size (Table 1).

**Table 1:** Classification of each human fixation.

	In AOI	In non-AOI
ViT with high attention (hit)	AOI_HIT	NONAOI_HIT
ViT with low attention (non-hit)	AOI_NONHIT	NONAOI_NONHIT

#### Procedure.

1. For each image in each group, we manually drew a polygon around the object of interest, generating the corresponding AOI mask.
2. For each fixation of each participant in each image, we determine:
  - whether the fixation point falls within an AOI or within a non-AOI region, and
  - whether the ViT exhibits attention above τ at that pixel (*hit*) or not (*non-hit*).
  This yields the following partition:
3. For each head *h*, we compute the hit rates (and non-hit rates) in the regions:
  $$HR_{AOI}=\frac{\text{AOI\_HIT}}{\text{AOI\_HIT}+\text{AOI\_NONHIT}},\;HR_{non}=\frac{\text{NONAOI\_HIT}}{\text{NONAOI\_HIT}+\text{NONAOI\_NONHIT}}$$
  and the *lift*, an advantage measure defined as the difference between the hit rates inside and outside the AOI:
  $$lift=HR_{AOI}-HR_{non}.$$
  Positive values of lift indicate a higher concentration of ViT attention in regions defined as relevant by the participants.
4. Finally, we conduct the statistical tests:
  (a) *Paired participant-level comparison (per head h).* For each participant *p*,
    $$HR_{AOI}^{\left(p,h\right)}=\frac{A_{p,h}}{A_{p,h}+B_{p,h}},\;HR_{non}^{\left(p,h\right)}=\frac{C_{p,h}}{C_{p,h}+D_{p,h}},$$
    where $A_{p,h}=\text{AOI\_HIT }$, $B_{p,h}=\text{AOI\_NONHIT }$, $C_{p,h}=\text{NONAOI\_HIT }$, and $D_{p,h}=\text{NONAOI\_NONHIT }$. We define $\Delta_{p,h}=HR_{AOI}^{\left(p,h\right)}-HR_{non}^{\left(p,h\right)}$ and test $H_{0}:𝔼[\Delta_{p,h}]=0$ using a paired *t*-test, reporting Cohen’s *d* as effect size. When appropriate, we also report the Wilcoxon signed-rank test as a robustness check.
  (b) For the contingency table
    $$\begin{array}{c} \left[\begin{array}{cc} \text{AOI\_HIT} & \text{AOI\_NONHIT}\\ \text{NONAOI\_HIT} & \text{NONAOI\_NONHIT} \end{array}\right] \end{array}$$
    we apply Pearson’s $\chi^{2}$ test without correction (if any expected count is < 5, we use Fisher’s exact test). We report the *p*-value and the *odds ratio* (with the Haldane–Anscombe correction, + 0.5) between the AOI/non-AOI and hit/non-hit categories, computed on counts aggregated by head.
  (c) All computations are performed separately for each object group, *basketry* and *jar*.

## Results

This section presents the results obtained from the previously outlined methodology. We separate the analysis into participant visualization and ViT model results, and subsequently discuss the fundamental problem of this research, focusing on the analysis of similarity and/or differences between ViTs and participants’ average perceptions.

### Sociodemographic information of participants

The experiment was conducted in the Neuroscience Laboratory of the School of Psychology at Universidad Adolfo Ibáñez (UAI) in Chile. In total, 30 participants provided informed consent to participate in the experiment. Each participant agreed to provide sociodemographic information, such as age, gender, and area of study (see Table 2). Of the total of 30 participants, 30% correspond to the female gender and 70% to the male gender, with an average age of 25.2 years (*SD* = 4.87) for the female gender and 24.3 years (SD = 3.62) for the male gender, with a total average of 24.6 years (*SD* = 3.97). Regarding the area of knowledge they ascribe to, 53.3% is associated with the engineering and sciences area (mathematics, data science, computer science), 36.7% with social sciences and arts (law, humanities, arts), and 10% with the business area (marketing, business administration).

**Table 2:** Sociodemographic information of participants.

	Count				Mean (years)				σ			
	BSN	SE	SSA	Σ	BSN	SE	SSA	μ	BSN	SE	SSA	All
Female	–	6	3	9	–	26,5	22,7	25,2	–	5,6	0,6	4,87
Male	3	10	8	21	27	24,4	23,1	24,3	7,8	1,1	3,7	3,62
Totals	3	16	11	30	27	25,2	23,0	24,6	7,8	3,5	3,1	3,97

Note: **BSN**: Business, **SE**: Sciences and Engineering, **SSA**: Social Sciences and Arts.

### Participant visualization

**Fig 6:**
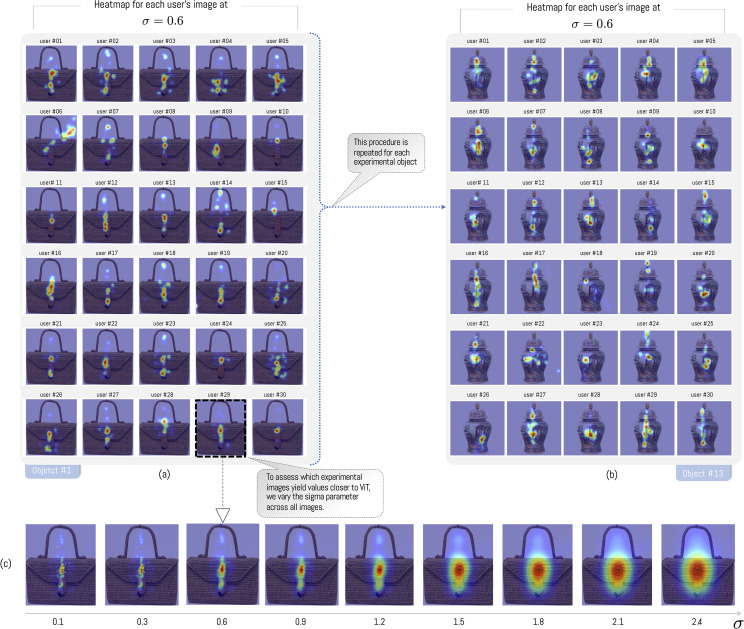
Heatmap analysis by each observer. (a) heatmap of each user for object #1 (basketry), (b) user’s gaze as the parameter σ increases, the greater the coverage area of the average vision. (c) heatmap of each user for object #13 (ginger jar).

The positions recorded by the eye-tracker enable the construction of a heat map that identifies the regions each user has maintained their gaze on. Thus, 600 heatmaps were obtained (30 participants by 20 observed objects). Although Pupil Capture software generates a position in terms of coordinates (*x*,*y*), the density can be subsequently modified through the estimation of a two-dimensional Gaussian distribution as a function of the points visualized on the screen (see Figs 5 and 6). This modification is performed by modifying the parameter σ, which defines the standard deviation of the Gaussian distribution (see change of σ in Fig 6b). As the parameter increases, the area and density of the zones where the study participants’ average gaze is centered also increase. On the contrary, when σ is low, we obtain isolated regions with low density (see example Fig 6 when σ=0.1). This process is repeated each time the parameter σ varies, totaling 40 variants of σ for each object, where:


σ∈{0.1,0.2,0.3,...,4.0}={0.1×k|k∈ℕ,1≤k≤40}

In this way, we obtained a total of 24,000 heatmaps with different σ values (600 heatmaps × 40 σ values), covering a relevant spectrum of variation. To visualize the evolution of σ for each user, Fig 6 illustrates both the individual images (30 participants) as well as the average attention maps for a specific σ value.

**Fig 7:**
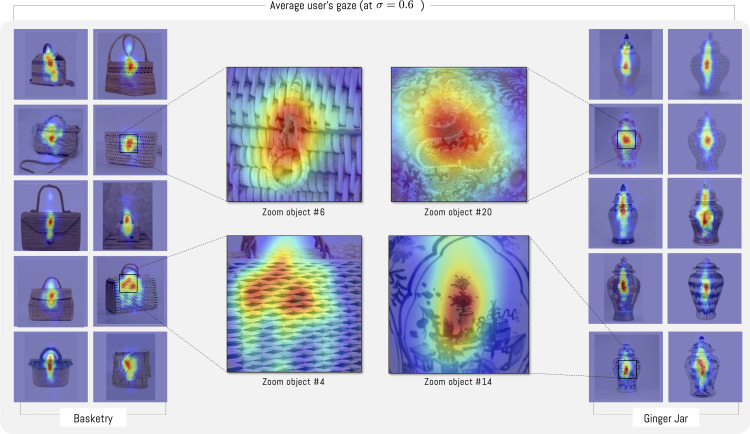
Density of users’ average gaze for each object for σ=0.6. Basketry: Zoom object #4: Detail of the region of an object without a buckle. Zoom object #6: Detail of buckle with longer observation time by users. Ginger Jar: Zoom object #14 vase symbol with the highest amount of observation.

In general, users focus their gaze on the center of objects (see Fig 6 for object #1). However, when performing this analysis for each object, large differences are observed between objects and their type. For example, in most basketry objects, users focus their gaze longer on the buckle, and do not pay attention to the object’s texture or its straps. In the case of jars, a vertical displacement of gaze is observed. As in the previous case, the observed data show textures and limited observations of ginger jar objects. This is mainly because observation is related to the task. Since the experiment is a free observation, there is no specific task the user must perform when encountering the observed objects. As a result of this process, we observe relevant differences between objects of the same category. For example, if we observe basketry objects #4 and #6, we notice that object #4 does not possess a buckle like the rest of the category (Fig 7, #4)

### Images generated by the ViT attention module

**Fig 8:**
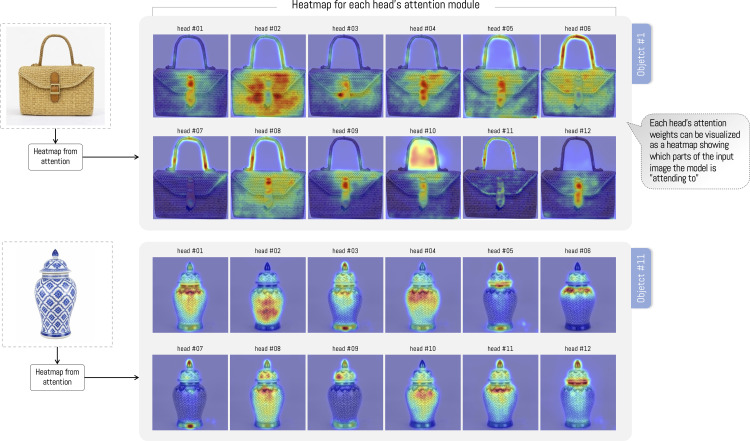
12 heatmaps generated by the ViT attention module, both for a basketry-type object and for a jar. Each of the 12 heatmaps represents part of the attention visualization within the algorithm. *Note: The craft figures shown are similar but not identical to the original images and are included for illustrative purposes only*.

**Fig 9:**
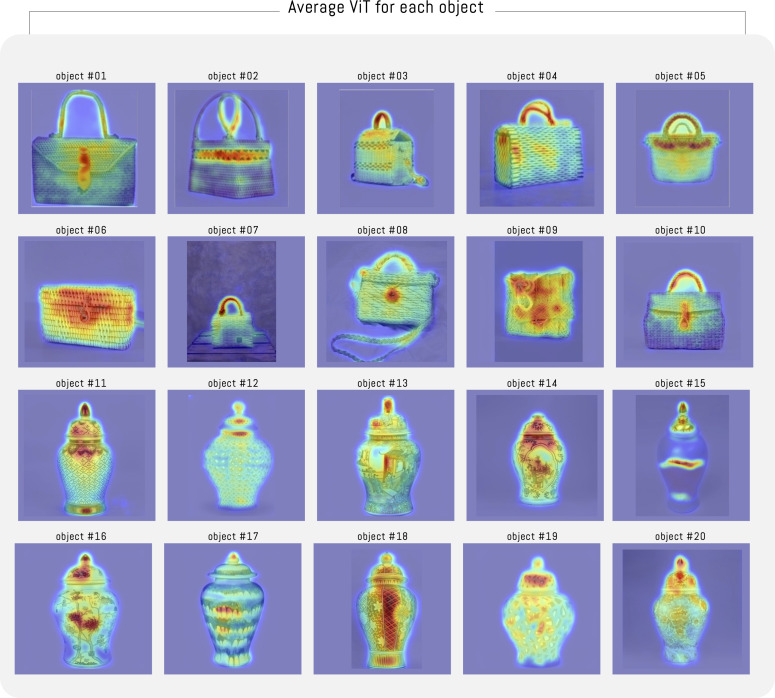
Average ViT for objects. Average heatmap of the 12 heads of the ViT attention module for each object in the experiment.

Fig 8 presents 12 heatmaps associated with the ViT attention module for objects #1 and #12. In the case of basketry-type objects, in some images, the ViT attention focuses on texture or on other specific zones of the object, such as the buckle or strap. Finally, in some cases, a mixture of both is presented (texture and buckle or strap). In the case of ginger jars, each head focuses on a zone of the object, regularly coinciding with the central zone and, in some cases, on the jar lid. Fig 9 presents the average of the 12 heatmaps for each object. The average shows that the attention module distributes its attention more evenly across the entire object. However, in the experiment images, we notice relevant differences between the categories used. In this sense, in all basketry objects, the buckle is marked by greater density, and the strap is over other zones. In the case of jars, the results are highly variable; most of the time, the ViT attention module focuses on the lid, and at other times on specific zones with textures and drawings of the jar. In some extreme cases, attention is concentrated on some characteristic of the object (see Fig 9-object#15).

### Differences between ViT and participant visualization

**Fig 10:**
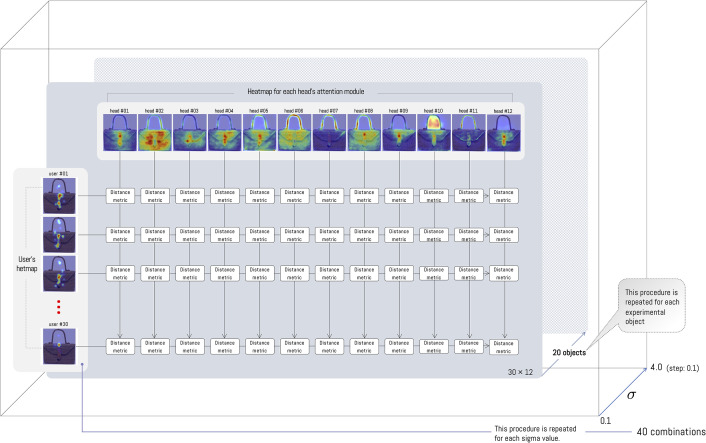
The distance between the participants and each ViT head is computed separately for each metric (KL, CC, SSIM, SIM). In this way, we estimate the distance between the 30 participants and the 12 heatmaps from the ViT module (30×12). This procedure is repeated for the 20 objects in the experiment (30×12×20), and since this distance is computed for a given σ, we evaluate multiple values with σ∈{0.1,0.2,0.3,…,4.0}. Thus, we obtain 30×12×20×40=288,000 combinations.

To measure the similarity or difference between the heatmaps produced by ViT and human attention, we compute distances using the four metrics described above, namely the Kullback–Leibler (KL) divergence, Structural Similarity (SSIM), Pearson’s Correlation Coefficient (CC), and Similarity (SIM). To apply each distance, the images to be compared must be normalized (min-max normalization to the [0,1] range). This process is performed for each image viewed by participants (Fig 7), for each value of σ, and for each ViT head (12 heatmaps per object) (see Fig 10). Regarding the distances, we computed the distance between each participant and each head (Fig 9). This step is relevant because we assume independence among experiment participants; that is, we retain individual distances with respect to the ViT.

The comparison process is performed independently for each of the 12 ViT attention heads against each of the 30 participants, across the 20 objects, and for each value of the σ parameter (40 settings), yielding a total of 12×30×20×40=288,000 evaluations. It is important to note that this number corresponds to the computation for a single metric; in this experiment, we analyze four metrics, and therefore we compute a total of 288,000×4=1,152,000 distances (see Fig 10). To simplify the analysis, we average the participant-level distances for each head, obtaining a 12×20×40 matrix. This average is not the same as averaging participants’ attention maps; instead, it is an average of the distances. This also allows us to compute the standard error of the estimate.

**Fig 11:**
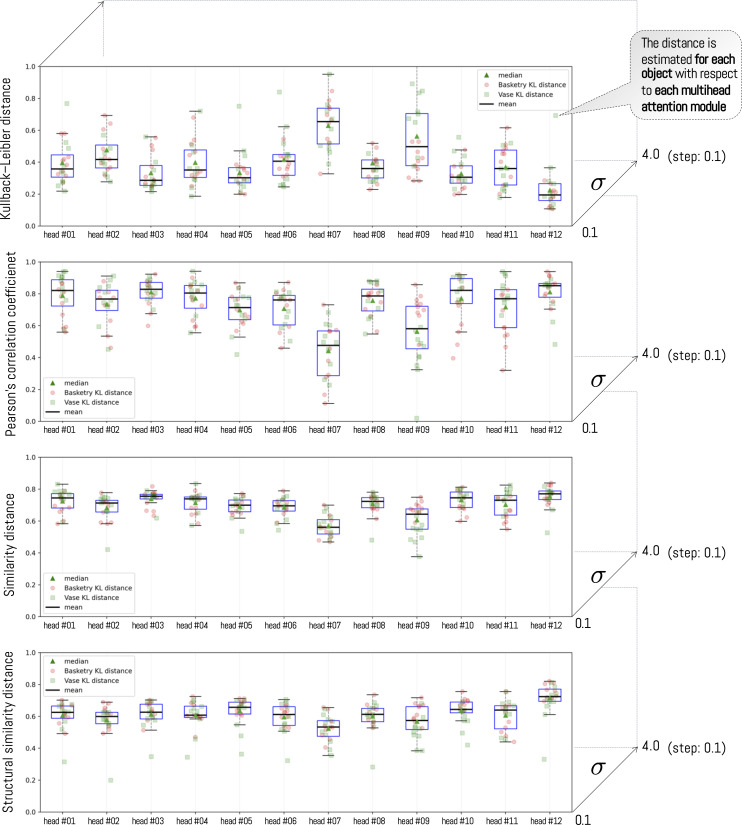
Each point in the box plot represents the distance between one of the 20 objects and each of the attention heads. In this example, the parameter σ is fixed at 2.6. Note that each mean corresponds to the average distance with respect to each head.

To visualize this distance for a given σ, the boxplot in Fig 11 presents the distance between each object versus each head (in this example, at σ=2.4) (for each distance metric). In general, distances between heads are similar. However, heads #7 and #9 consistently have a greater or smaller distance than the other heads. This means these heads are furthest from the participants’ view. Regarding the types of objects we use, we can observe differences within the same head across categories. This means that heads can have a greater or lesser distance for some classes of objects than for others. For example, head #1 consistently has a smaller distance for Vase-type objects than for Basketry-type objects. The opposite effect occurs in head #9 where the head has better performance for basketry-type objects. This means that human vision behaves differently from the ViT, not that the ViT necessarily produces different results depending on the type of object.

**Fig 12:**
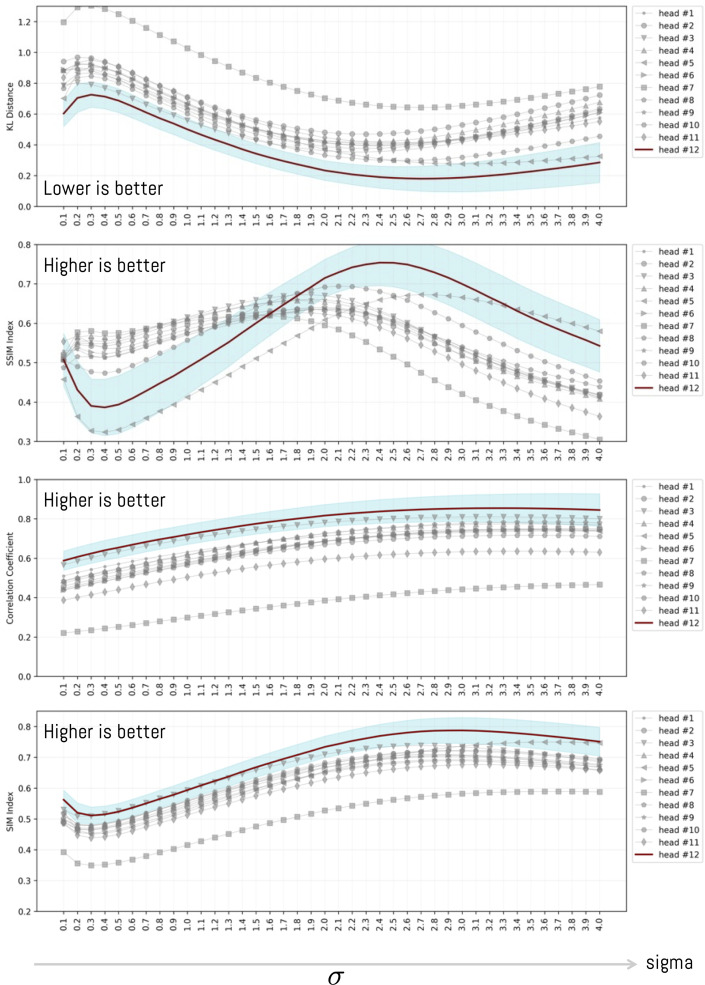
Variation of distance as the value of σ increases for each head. Each distance is computed as the average distance between the average visualization across all objects and each ViT head. Across all metrics, head 12 attains the value closest to human attention. However, as σ increases, the standard error (shown in light blue) also increases.

To analyze in detail the effect of varying the parameter σ, we sweep σ from 0.1 to 4.0 in steps of 0.1. For this purpose, we use the mean value obtained for each box plot in Fig 11. In this way, we examine the average behavior as the parameter increases. The outcome of this comparison is shown in Fig 12, separated by head and metric. From the plot, it can be seen that although each head may exhibit a different pattern, a general trend emerges: there exists an optimal value for which the distance between a head and the participant-level average distance metric is minimized (or maximized, depending on the metric). The results consistently show the same pattern, with head #12 consistently the closest to the participants’ visualization. In this sense, the KL and SSIM metrics yield very similar results despite their different formulations. For KL, the best value corresponds to the minimum, whereas for SSIM the best value corresponds to the maximum; in both cases, the best values occur around σ=2.4 and σ=2.6, respectively. In addition, the CC and SIM metrics display a similar behavior, and in this case, performance does not improve beyond σ=2.7.

**Fig 13:**
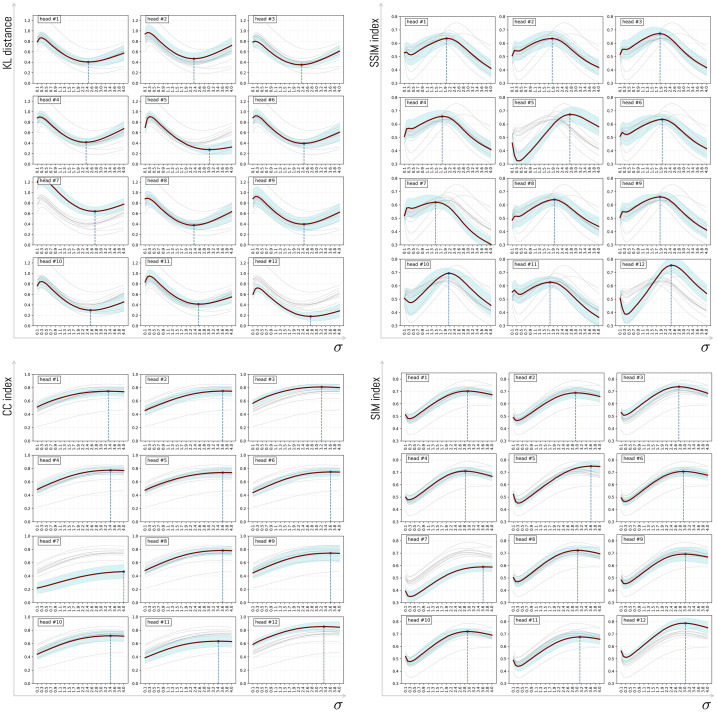
Variation of distance as the value ofσ increases for each head. Each distance is computed as the average distance between the average visualization across all objects and each ViT head. The blue region corresponds to the 95% confidence interval.

Fig 13 shows the same result as above, but additionally estimates confidence intervals for each head as σ increases. In this sense, experimentally we observe that heads #3, #10 and #12 are more similar to the average visualization of people and that, in addition, they possess the lowest variance among the different compared objects. As the parameter σ begins to increase. When sigma increases, and at the same time, the standard error of the sample (see blue background under each line). It is worth noting that some of the heads do not have results close to the average visualization of participants. This is the case of head #7 and head #9, where the dispersion is greater with respect to the other heads. This result had already been analyzed previously for the specific case of σ=2.6 (see Fig 11).

When analyzing the variation of σ over a range of values, the same performance is observed consistently. This result aligns with previous research, which shows that ViT heads do not necessarily fix attention on the same regions as a human visualization would.

**Fig 14:**
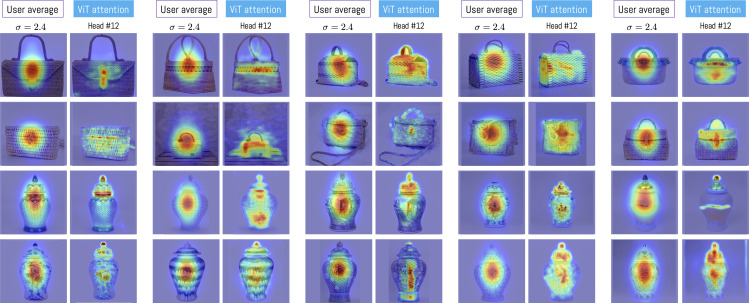
Comparison between each average visualization with respect to head #12 of the ViT attention module. In the case of average visualization, a value of σ=2.4 has been considered.

To simplify the analysis, Fig 14 illustrates the differences between the participants’ average visualization and head #12 of the attention module for each of the 20 objects in the experiment. It can be observed that the participants’ average visualization tends to concentrate toward the center of the image; in contrast, ViT head #12 highlights specific regions of the object, which in some cases coincide with the participants’ average visualization. This example illustrates how one of the 12 heads tends to approximate the average visualization of the experiment participants. It is important to note that in our experiment, we did not compute an average image; it is shown only to support the interpretation that the participants’ average visualization is not necessarily aligned with the ViT visualization.

**Fig 15:**
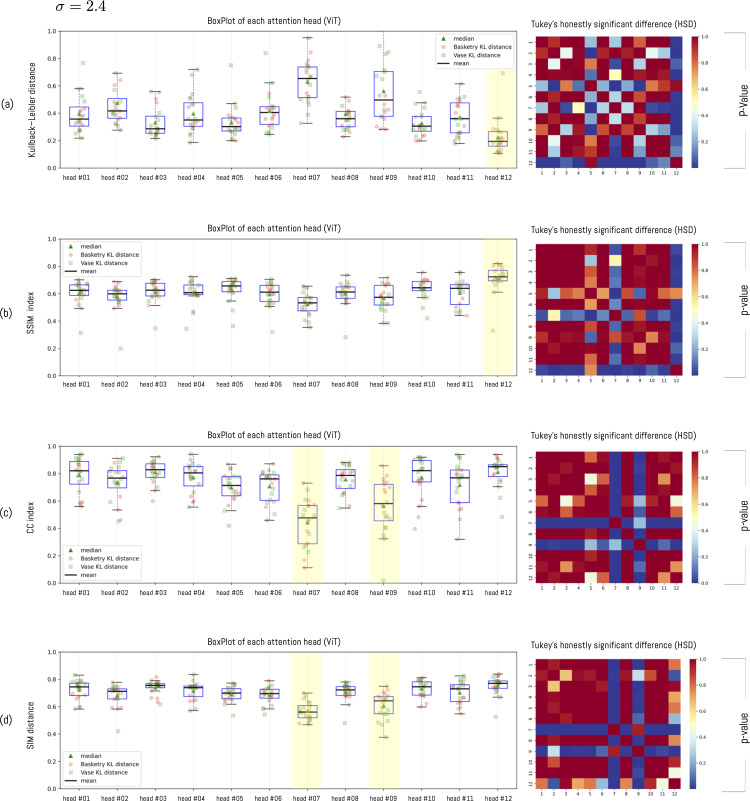
Tukey honestly significant difference (HSD) with different σ. Difference between Tukey Honestly Significant Difference (HSD) to measure the difference in means between attention module heads with three variants of σ.

To analyze the distance relationship between the different heads, we employed the Tukey Honestly Significant Difference (HSD) test after checking with an ANOVA test that there is significant difference between the means (see Fig 15). For this we fixed the value of σ=2.4 and applied the test between all heads. The results indicate that heads #7, #9 and #12 are statistically different from the rest of the heads, given that their p-values in most cases are less than 5%. These results, together with those presented in Fig 12, allow us to affirm that heads #7 and #9 are those with the greatest distance to human visualization and head #12 with the smallest distance. This means that heads #7 and #9 are very different from the participants’ visualizations, whereas head #12 is the closest. However, this pattern does not hold across all metrics. Only for KL and SSIM do we observe a statistically significant difference for head #12 relative to the others. By contrast, although head #12 attains the highest value for the CC and SIM metrics, the difference is not statistically significant.

**Fig 16:**
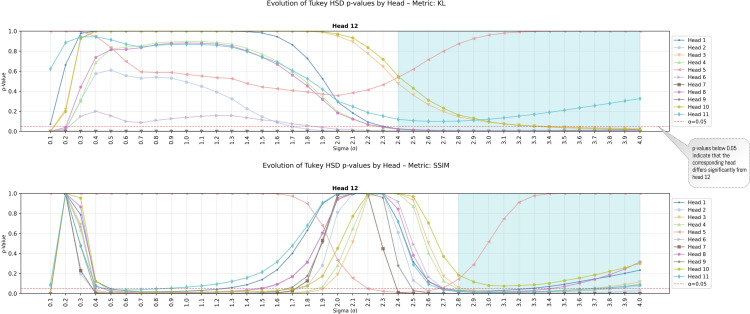
Behavior of the *p*-value for head #12 according to the HSD test. The analysis was performed only for the KL and SSIM metrics, as they exhibit a statistically significant difference.

To further examine the effect of the parameter σ on the KL and SSIM metrics, we analyzed the behavior of the *p*-value from the HSD test. The results show that, beyond a certain threshold, most heads yield values below 5% (Fig 16) when compared against head #12. This indicates that, for sufficiently large σ, head #12 is statistically different from the others. To perform this analysis, we applied the HSD test for each possible value of σ and evaluated its behavior by measuring the average distance of each head relative to the others. We focused our analysis exclusively on head #12 because, according to the previous results (Fig 12), it is consistently different from the remaining heads.

### Statistical analyses in Areas of Interest (AOI)

Following the procedure described in the subsection “Statistical analysis of areas of interest (AOI)”, for each ViT head *h* we computed, per participant and for each image group (*basketry* and *jar*), the hit rates inside and outside the AOI using the threshold τ defined per image and per head. From these rates, we computed the *lift* $\left(HR_{AOI}-HR_{nonAOI}\right)$ and carried out the specified statistical tests.

**Fig 17:**
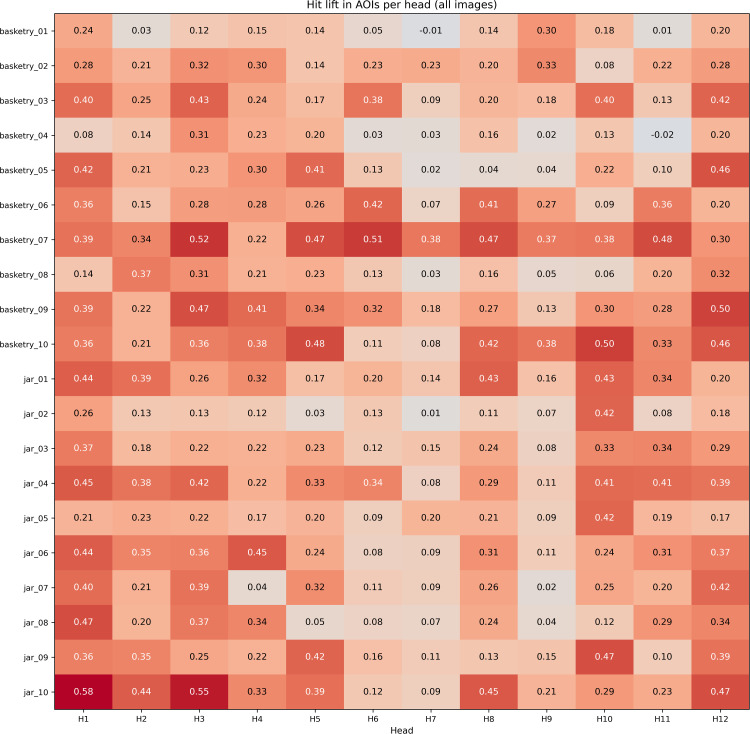
Heatmap of *lift* by head and image. Each cell reports $HR_{AOI}-HR_{nonAOI}$. Color highlights the magnitude of the effect. The threshold τ was defined per image and per head, as described in the evaluation section.

As a global overview before the semantic-domain breakdown, Fig 17 shows, across all images, the *lift* $HR_{AOI}-HR_{nonAOI}$ per head. Values are annotated in each cell; warmer colors indicate a larger advantage within the AOI.

The head-wise *lift* heatmap (Fig 17) shows a predominance of positive values across columns, indicating that ViT activations are more frequently concentrated within AOIs than in their complementary regions; several heads maintain high and stable values across images, whereas others exhibit more moderate advantages.

To analyze behavior by semantic domain, we now report results stratified by object type. Each table summarizes, by head:

- The mean and standard deviation of *HR*_AOI_ and *HR*_non–AOI_, computed from participant-level rates.
- The *lift* difference in percentage points: ${\text{lift}}_{pp}=100\cdot (HR_{\text{AOI}}-HR_{\text{non-AOI}})$.
- The head-wise association test ($\chi^{2}$ or Fisher), the *p*-value, and the *odds ratio* (OR) with the Haldane–Anscombe correction.

As shown in Table 3, all heads exhibit significantly higher hit rates within AOIs than in non-AOI regions (*p* < 0.0001 in all association tests). The largest differences are observed for H12 and H3 (+33.1 and +33.0 pp, respectively), followed by H1 (+30.3 pp) and H5 (+28.0 pp), whereas H7 shows the most moderate effect (+10.4 pp).

**Table 3:** AOI analysis results for the *basketry* set.

Head	Hit rate	Hit rate	lift (%)	Test	p-value	Odds
	AOI	nonAOI		$\chi^{2}$		Ratio
12	33.1 ± 21.5	0.0 ± 0.0	33.1	40271.2	<0.0001	90741.3
3	33.3 ± 21.0	0.3 ± 1.8	33.0	36551.6	<0.0001	79.7
1	30.7 ± 20.6	0.4 ± 2.0	30.3	32836.8	<0.0001	61.8
5	28.8 ± 20.7	0.8 ± 6.7	28.0	29892.9	<0.0001	64.1
4	27.0 ± 17.8	0.0 ± 0.1	27.0	31031.4	<0.0001	3579.0
8	24.3 ± 19.6	0.1 ± 0.9	24.1	23721.1	<0.0001	505.8
10	23.1 ± 20.2	0.0 ± 0.2	23.0	23581.9	<0.0001	382.4
6	22.6 ± 20.5	0.1 ± 0.8	22.5	21201.9	<0.0001	99.7
2	20.9 ± 17.4	0.0 ± 0.0	20.9	21096.3	<0.0001	44832.6
9	21.0 ± 18.5	0.5 ± 6.3	20.6	20452.5	<0.0001	245.1
11	22.2 ± 19.9	2.0 ± 8.7	20.2	17874.9	<0.0001	19.2
7	13.0 ± 18.0	2.7 ± 11.5	10.4	8083.9	<0.0001	11.8

The *odds ratios* confirm the magnitude of these effects: H12 (OR = 90 741.3), H2 (OR = 44 832.6), and H4 (OR = 3 579.0) stand out, indicating that a hit within the AOI is tens to thousands of times more likely than in non-AOI regions for the best-aligned heads.

Mean hit rates within AOIs range from 13.0 % to 33.1 %, compared with 0.0–2.7 % in non-AOI regions. The standard deviations (±17–21 pp) reflect expected heterogeneity across images and participants; nevertheless, the enrichment pattern AOI > non-AOI remains consistent.

In summary, the *basketry* set exhibits positive and highly significant *lift* across all heads (Table 3). H12, H3, H1, and H5 concentrate the largest *lifts* (≥28 pp) and extreme OR values (up to $9.1\times 10^{4}$), consistent with human foci on buckles and high-contrast textures.

As shown in Table 4, all heads likewise exhibit significantly higher hit rates within AOIs than in non-AOI regions (*p* < 0.0001 in all tests). The largest differences are observed for H1 (+39.9 pp), H10 (+33.7 pp), H12 (+32.2 pp), and H3 (+32.0 pp), whereas H9 and H7 show the most moderate effects (+10.3 and +10.2 pp).

**Table 4:** AOI analysis results for the *jar* set.

Head	Hit rate	Hit rate	lift (%)	Test	p-value	Odds
	AOI	nonAOI		$\chi^{2}$		Ratio
1	40.0 ± 19.7	0.0 ± 0.5	39.9	35577.9	<0.0001	818.6
10	33.7 ± 20.1	0.0 ± 0.0	33.7	28064.5	<0.0001	60954.2
12	32.6 ± 18.4	0.4 ± 3.0	32.2	25701.5	<0.0001	187.0
3	32.3 ± 19.5	0.3 ± 3.6	32.0	26215.3	<0.0001	2099.7
2	28.8 ± 19.6	0.0 ± 0.0	28.8	22587.7	<0.0001	48249.7
8	26.8 ± 18.7	0.0 ± 0.1	26.7	20154.2	<0.0001	3887.8
11	26.2 ± 17.2	1.2 ± 8.4	25.0	19354.3	<0.0001	239.4
4	24.7 ± 19.7	0.5 ± 6.9	24.2	18108.0	<0.0001	498.3
5	27.7 ± 17.5	3.6 ± 16.9	24.1	8413.4	<0.0001	3.6
6	15.6 ± 16.3	1.2 ± 8.4	14.4	9973.1	<0.0001	78.7
9	11.9 ± 14.6	1.6 ± 8.7	10.3	6853.9	<0.0001	44.6
7	13.0 ± 12.8	2.9 ± 12.2	10.2	7209.6	<0.0001	21.3

The *odds ratios* confirm the magnitude of these effects: H10 (OR = 60 954.2), H2 (OR = 48 249.7), and H8 (OR = 3 887.8) stand out, indicating that a hit within the AOI is tens of thousands of times more likely than in non-AOI regions for the best-aligned heads.

Mean hit rates within AOIs range from 11.9 % to 40.0 %, whereas in non-AOI regions they lie between 0.0 % and 3.6 %, depending on the head. The standard deviations (± 12.8–20.1 %) reflect heterogeneity across images and participants, as expected under free viewing of jars. Part of the extreme OR values is associated with near-zero non-AOI rates; however, the systematic enrichment within AOIs remains consistent.

In summary, the *jar* set shows positive and statistically significant *lift* across all heads. H1, H10, H12, and H3 concentrate the largest fractions of hits within AOIs, whereas H9 and H7 present the most moderate effects. The elevated OR values reinforce this pattern even after applying the Haldane–Anscombe correction to head-aggregated counts.

For the *jar* set (Table 4), results maintain the same absolute significance trend (*p* < 0.0001). Head H1 leads in *lift* (+39.9 pp), followed by H10, H12, and H3 (≥32 pp). Again, extreme OR values are observed for heads such as H10 (OR = 60 954.2) and H2 (OR = 48 249.7), partly explained by near-zero non-AOI rates. The standard deviations (±12.8−−20.1%) reflect the intrinsic variability in participants’ visual exploration of this object type.

In summary, Tables 3 and 4 confirm systematic enrichment within AOIs for both domains. Heads H12, H1, and H3 consistently stand out as the most aligned with human observers, capturing the main effect together with domain-specific complementary heads (H5 in *basketry* and H10 in *jar*). The robustness of this pattern ($\chi^{2}\geq 6\;853$) suggests these heads as strong candidates for aesthetic-interest prediction tasks or for implementing AOI-guided loss functions in artisanal design models.

## Discussion

Computer vision has made significant advances in developing attention mechanisms inspired by human vision [59]. Thus, vision transformers have demonstrated high potential in this field for different tasks, equaling or even surpassing Convolutional Neural Networks (CNN), which present limitations compared to human attention [54,60,61]. Our research continues to deepen the relationship between human attention and that performed by unsupervised ViTs [53,62]. For this, hypotheses were established that there are no significant differences between the attention produced by a ViT model and human attention (Hyp.1), so it can be an applicable technology in the design and creation process of artisanal products for detecting elements with greater aesthetic attractiveness (Hyp.2). To respond to the hypotheses, objectives were established to statistically determine the correlations between both attention mechanisms and analyze visual interest points in artisanal objects through these attentions.

The methodology established to achieve the objectives was organized in three stages (data preparation, modeling and evaluation), where a dataset of images of two typologies of artisanal objects was constructed, 10 bags made through basketry and 10 ginger jars, with different stylistic characteristics where, in the case of bags made with basketry, for their selection structural unity was sought, with polygonal forms predominating, with small curvilinear contrasts in handles or closure clasps. The bags have similar textures and colors, including some differentiating examples with small details in different colors and materials to draw attention to variations between images. In the case of ginger jars, curvilinear forms and verticality predominate. These crafts feature different aesthetic elements in their decoration, such as floral motifs or naturalistic representations, as well as textures resulting from decorative or structural frameworks.

These artisanal objects were visually evaluated by 30 people (9 women and 21 men), with an average age of 24.6 years (SD = 3.97), using an Eye Tracker that recorded participants’ observations. Using the obtained data, 600 heatmaps were generated, normalized and averaged using a two-dimensional Gaussian distribution with varying σ values, allowing subsequent comparison with the ViT results.

The results from participants’ viewing in the experiment align with expectations based on the objects’ stylistic characteristics [63,64]. In the case of bags with closure buckles, attention is concentrated, while objects without this element disperse attention across surfaces, especially on textures that differ from the general structure. This occurs because the set includes differentiating forms that help visual attention. In this case, the circular closures create a contrast with the bag structures’ general rectilinear forms. In objects viewed without this element, textures that differ from the general set exhibit distinct framework and color characteristics that draw greater visual attention. In contrast, ginger jars elicit attention that ascends and descends, as reflected in their morphology. As with basketry craft objects, central attention predominates, which coincides with previous studies on aesthetic preferences and spatial composition [65], although deviated attentions are detected when the object possesses some decorative element, especially images of ginger jars that have floral motifs and represented people (Fig. 7), which is consistent with previous works on Object-based visual attention [66,67].

Most of the images used in the experiment do not have a background, since this context can be distracting by introducing semantic content [68]. Only one image of a basketry bag with a background and another element (a table where the object rests) has been preserved to analyze whether these elements distract from attention. The results do not show significant human attention to these elements.

The same image dataset was analyzed using the Vision Transformer (ViT), pre-trained with DINO (Self-Distillation with NO Labels). This architecture generates 12 independent distributions (heads). This implies the creation of 240 heat maps (12 for each craft object). As observed in Fig 9, the attention performed by ViT is deployed over the entire surface of the object, coinciding with previous research that indicates the tendency toward globalized attention in images [62]. This is consistent with the research by [53], who indicate that ViTs do not perform selective attention but rather group elements by element characteristics, acting as a horizontal relaxation of labeling and bottom-up processing in human attention. However, unlike our work, the researchers do not collect and use data on human attention. This allows us to observe certain similarities, as both ViT and human observers of basketry bags focus on buckles when they are present. However, differences are observed in attention to jars, where ViT does not show the same verticality as the experiment participants.

To better understand the results, the attention produced by each ViT head was analyzed, following previous research such as [69] and [70], aiming to better understand the model’s internal functioning. For this, normalisation (normMixMax) was performed on each average image of participants and on each ViT head, and subsequently, the parameter σ was systematically varied across σ∈{0.1,0.2,0.3,…,4.0} (see Fig 6).

To reinforce the robustness of our findings, we performed divergence analyses using Kullback-Leibler (KL), structural similarity index (SSIM), Pearson’s correlation coefficient (CC), and similarity (SIM). This multi-metric approach aligns with established saliency benchmarking practices, which recommend using multiple complementary metrics since each captures different properties of spatial distributions. As Bylinskii et al. [71] demonstrated, different metrics can rank saliency models differently depending on how they handle false positives, false negatives, and spatial deviations. The combination of these four metrics enabled us to compute 1,152,000 distance evaluations (30 participants × 12 heads × 20 objects × 40 σ values × 4 metrics), providing comprehensive coverage of the parameter space.

The results show consistent patterns across all metrics: head H12 emerged as the closest to the participants’ visualization regardless of the metric employed, while heads H7 and H9 showed the greatest divergence (Fig 12). This consistency across methodologically distinct metrics strengthens confidence in the finding. Statistically significant differences (p≤0.05) between head H12 and the remaining heads were observed for KL and SSIM metrics when applying the Tukey HSD test Figs 15 and 16). For CC and SIM, although H12 achieved the highest values, the differences did not reach statistical significance. This pattern is consistent with Kümmerer et al.’s [72] finding that no single saliency map performs optimally across all metrics, suggesting that KL and SSIM may be more sensitive to the specific distributional differences characterizing human-ViT attention alignment.

The results also reveal that heads H3, H11, and H12 are the most consistent in their correlation with human attention according to all distance metrics, while heads H7 and H9 show the greatest dispersion. This result is partially consistent with the study by Yamamoto et al. [62], who found that attention heads in DINO-trained ViTs autonomously differentiate into distinct functional clusters, with approximately 20% focusing on key points within figures—a pattern that may correspond to the aligned heads identified in our study.

To understand in detail the effect of variation in the parameter σ, we analyzed behavior across all metrics. The results show that the optimal value is found at σ=2.4±0.03 for KL and SSIM, with head H12 being the closest to the average attention of participants and maintaining consistency across all analyses (Fig 14). The analysis of p-value behavior across σ values (Fig 16) confirmed that this head maintains statistical distinctiveness from other heads beyond a threshold of approximately σ=1.5.

Beyond correlational measures based on spatial distributions, we implemented an Areas of Interest (AOI) analysis to determine whether ViT attention concentrates on objects themselves rather than background regions. It is important to note that in our implementation, AOIs were defined by manually drawing polygons around each complete object, distinguishing object regions from background but not differentiating sub-object components (e.g., buckle vs. strap in basketry, or lid vs. body in jars). This approach follows established practices in eye-tracking research where AOIs represent regions of semantic significance [73], though we acknowledge that finer-grained AOI definitions could reveal additional patterns [74].

The results revealed that all 12 attention heads exhibited significantly higher hit rates within object AOIs than in background regions (*p* < 0.0001 for all heads in both object categories, Tables 3 and 4). This finding demonstrates that ViT attention systematically focuses on objects rather than being randomly distributed or drawn to background elements—a non-trivial result given that the DINO training objective does not explicitly encourage object-focused attention.

For the basketry set (Table 3), heads H12 and H3 showed the largest lift values (+33.1 and +33.0 percentage points, respectively), indicating that when humans fixated within the object region, these ViT heads were approximately 33 percentage points more likely to show high activation compared to background regions. Head H1 also demonstrated strong performance (+30.3 pp). For the jar set (Table 4), head H1 achieved the highest lift (+39.9 pp), followed by H9 (+33.7 pp) and H12 (+32.2 pp).

The chi-square statistics confirm the robustness of these associations ($\chi^{2}\geq 6,853$ across all tests). Regarding the odds ratios, some heads exhibited extremely high values (e.g., OR = 90,741 for H12 in basketry). However, these extreme ORs should be interpreted with caution, as they result from near-zero hit rates in background regions (e.g., 0.0±0.0 for H12 non-AOI). While the Haldane-Anscombe correction (+0.5) was applied, such extreme ratios primarily indicate that these heads rarely activate strongly in background regions, rather than providing precise quantitative estimates of effect magnitude. The lift values in percentage points provide a more interpretable measure of the attention-concentration effect.

A key finding of this research is the convergence of evidence from the metric-based and AOI analyses. Heads H12, H1, and H3 consistently emerge as the most aligned with human observers across both analytical approaches and both object domains:

- H12: Lowest distance to human attention in metric analysis; highest lift in basketry AOI analysis (+33.1 pp); strong performance in jar AOI analysis (+32.2 pp)
- H1: Strong metric performance; highest lift in jar AOI analysis (+39.9 pp); strong basketry performance (+30.3 pp)
- H3: Consistent metric alignment; second-highest lift in basketry (+33.0 pp); strong jar performance (+32.0 pp)

This convergence across methodologically distinct approaches—one measuring global distributional similarity, the other measuring object-focused concentration—provides robust evidence that these heads capture aspects of visual attention that align with human perception.

Conversely, heads H7 and H9 consistently showed weaker alignment in both analyses. Head H7 exhibited the lowest lift values in both object categories (+10.4 pp basketry; + 10.2 pp jars), and H9 showed similarly modest performance. One possible explanation is that these heads may be specialized for features relevant to the self-supervised training objective (e.g., texture patterns for contrastive learning) rather than the salient features that attract human aesthetic attention. Alternatively, these heads may encode background context or global scene statistics. Further investigation through attention ablation studies [73] could clarify whether these heads serve essential functions for model performance despite their divergence from human patterns.

The AOI analysis revealed domain-specific patterns that merit discussion. While H12, H1, and H3 showed strong alignment across both object categories, complementary heads emerged for each domain: H5 exhibited enhanced performance for basketry objects (+28.0 pp lift) compared to its jar performance (+24.1 pp), whereas H10 showed the opposite pattern, performing better for jars (+33.7 pp) than for basketry (+23.0 pp).

This domain specificity may reflect different visual processing strategies for distinct morphological categories. Basketry items, characterized by high-contrast buckles against uniform woven textures, may preferentially activate heads specialized in detecting isolated salient points. Jars, with their distributed decorative patterns and curvilinear contours, may engage heads that attend to broader figural regions or vertical structural elements. This interpretation aligns with Yamamoto et al. [62] finding of three functionally distinct head clusters in DINO-trained ViTs: one focusing on key points within figures (20% of heads), one distributing attention over entire figures (60%), and one attending primarily to background (20%).

These converging lines of evidence provide nuanced support for our hypotheses. Regarding Hyp.1 (no significant differences between ViT and human attention): The results partially support this hypothesis for specific attention components. While the global attention distributions of most ViT heads differ significantly from human attention patterns (as evidenced by heads H7 and H9), heads H12, H1, and H3 demonstrate sufficient alignment to suggest that certain ViT components approximate human attentional behavior. The statistical tests confirm that H12 is significantly different from other heads (p≤0.05, Tukey HSD), indicating it occupies a distinct position closer to human attention. However, we cannot claim that even H12 is statistically indistinguishable from human attention—rather, it is significantly more aligned than other heads.

Regarding Hyp.2 (ViT applicability for detecting aesthetic interest zones): The evidence more strongly supports this hypothesis. The systematic concentration of ViT attention within object regions (lift values +10 to +40 pp across all heads, all *p* < 0.0001) demonstrates that ViT attention reliably distinguishes objects from backgrounds in ways consistent with human visual attention. The identified heads with the highest alignment (H12, H1, H3) represent promising candidates for implementation in automated aesthetic evaluation systems. This aligns with emerging applications of attention mechanisms in design evaluation [75], where AI-based tools are being developed to predict consumer visual interest before product launch.

The practical implications of these findings extend to artisanal product design, where understanding consumer visual attention is crucial for market success. Previous research has established correlations between visual attention, aesthetic preference, and purchase intention [23–26], suggesting that attention patterns may serve as proxies for consumer interest. Our findings indicate that specific ViT attention heads could provide designers with rapid, automated feedback on which product features are likely to attract consumer gaze.

For basketry products, heads H12 and H3 could be used to verify that design elements intended to be focal points (such as buckles or decorative contrasts) actually generate high attention activation. For ceramic products like ginger jars, heads H1 and H10 may be particularly useful for evaluating whether decorative elements and overall form successfully capture attention in patterns similar to human observers.

However, we emphasize that these tools should complement rather than replace human judgment in design evaluation. The AOI analysis demonstrates object-level attention alignment, but does not guarantee that ViT and humans attend to the same sub-object features for the same cognitive reasons. The demonstrated alignment provides a foundation for further development of ViT-based design tools, but practical applications should be validated through additional studies linking ViT attention predictions to actual consumer behavior metrics.

## Limitations and future directions

Among the main limitations of this research, the reduced number of participants in the experiment stands out, so the sample cannot be considered representative, and this makes it difficult to perform experiments taking into account sociodemographic data and gender perspective. Therefore, we intend to continue increasing the sample in the future. Additionally, we intend to expand the experiment to other geographical and cultural contexts, since, like aesthetic experience, the cultural context of observers determines visual attention along with other factors, such as age, gender, educational level or field of study [76–78]. This is why, in this research, such information has begun to be compiled but has not been included in the methodology due to its limited relevance (e.g., note that, among the participants in this research, the number of men is significantly higher than the number of women to make a comparison). Undoubtedly, in the future, these demographic data and the incorporation of gender perspective will allow greater thoroughness in analyses and reach more significant conclusions, where we can hypothesize if any gender is closer to attention performed with ViT, or if there are differences between observers with different levels of education or areas of knowledge, making a comparison equally with ViT.

The Areas of Interest analysis validated object-versus-background attention alignment but employed coarse granularity, defining AOIs around complete objects rather than differentiating semantic sub-components such as buckles, straps, and body in basketry, or lids, decorations, and bases in jars. This limits conclusions about whether ViT and humans attend to the same specific features within objects. Implementing hierarchical AOI structures [74] that enable analysis at multiple semantic levels—from whole-object to specific decorative elements—would clarify whether the global alignment observed extends to fine-grained aesthetic features.

The results of this research reveal greater correlation between certain ViT heads and human attention, but this finding requires deeper analysis to understand the model’s mechanisms. For example, the importance of each head could be studied following Michel et al. [70], who found that disabling certain heads did not significantly affect performance. Using this methodology, we could determine whether the heads that showed significance in our experiment are actually fundamental to the attention model.

Another future line of action involves analyzing the correlation between purchase intention and visual attention patterns for the same product. This experimental approach would require observers to indicate their purchase intention while viewing objects, with simultaneous recording of response times and eye fixations [25,26,79,80]. Such an approach would enable us to determine more precisely whether aesthetic interest serves as a determining factor in purchase decisions [81].

## Conclusions

This study examined the alignment between Vision Transformer (ViT) attention and human visual attention during aesthetic evaluation of artisanal objects, combining multi-metric distance analysis with Areas of Interest (AOI) validation.

Three principal findings emerge. First, across 1,152,000 metric computations (KL, SSIM, CC, SIM), head H12 consistently demonstrated the strongest alignment with human attention (p≤0.05, Tukey HSD at σ=2.4), with heads H1 and H3 also showing robust performance. Second, all 12 ViT heads concentrated attention significantly more within object regions than background areas ($p<0.0001\;\chi^{2}\geq 6,853$), with H12, H1, and H3 achieving lift values of +30 to +40 percentage points. Third, the convergence of evidence across both analytical approaches—and across both object domains—identifies these three heads as consistently aligned with human observers, while revealing domain-specific patterns (H5 for basketry, H10 for jars).

Regarding our hypotheses: Hyp.1 (no significant difference between ViT and human attention) receives partial support—specific heads approximate human attention while others diverge substantially. Hyp.2 (ViT applicability for aesthetic interest detection) receives stronger support, as the robust object-level concentration and identification of aligned heads demonstrate potential for design evaluation applications.

In conclusion, self-supervised Vision Transformers develop attention mechanisms that can approximate human visual attention in aesthetic contexts. These findings contribute to understanding the intersection of artificial and human visual processing, while establishing a foundation for practical applications in artisanal product design that should be validated through behavioral studies linking attention predictions to consumer outcomes.
